# Coral reef degradation is not correlated with local human population density

**DOI:** 10.1038/srep29778

**Published:** 2016-07-20

**Authors:** John F. Bruno, Abel Valdivia

**Affiliations:** 1 Department of Biology, The University of North Carolina at Chapel Hill, Chapel Hill, NC, 27599-3280, USA; 2 Center for Biological Diversity, 1212 Broadway Street, Oakland, CA 94612, USA.

## Abstract

The global decline of reef-building corals is understood to be due to a combination of local and global stressors. However, many reef scientists assume that local factors predominate and that isolated reefs, far from human activities, are generally healthier and more resilient. Here we show that coral reef degradation is not correlated with human population density. This suggests that local factors such as fishing and pollution are having minimal effects or that their impacts are masked by global drivers such as ocean warming. Our results also suggest that the effects of local and global stressors are antagonistic, rather than synergistic as widely assumed. These findings indicate that local management alone cannot restore coral populations or increase the resilience of reefs to large-scale impacts. They also highlight the truly global reach of anthropogenic warming and the immediate need for drastic and sustained cuts in carbon emissions.

Coral reef communities have been substantially altered by human activities over the last several decades. The abundance and diversity of fishes and reef-building corals have decreased dramatically and the dominant benthic taxa on many reefs are now sponges, gorgonians, and macroalgae^1^,^2^,^3^,^4^,^5^,^6^. Such reef degradation directly affects vulnerable coastal communities in numerous ways, including the loss of income from fishing and tourism and reduced coastal buffering of storms as reefs die and erode away^7^,^8^.

Coral reef degradation is thought to be driven by both local and global factors, some independent and others interacting. For example, the increase in fleshy macroalgae is due to a combination of reduced herbivory, increased nutrient input, and reduced occupancy of the seafloor by coral (AKA “coral cover”), which alleviates competition and opens benthic substrata for colonization by other benthic taxa^9^,^10^,^11^. The underlying causes of “phase shifts” from coral to macroalgal dominance are also numerous and include diseases and bleaching that kill corals, fishing that removes herbivores, and increased runoff from coastal erosion, agriculture, and human sewage^9^,^12^,^13^. All these processes have complex and deeper ultimate causes including climate change, poverty, human population growth, poor governance, and inadequate local management.

Likewise, fish populations are declining due to both fishing and coral mortality that leads to reduced habitat complexity^14^,^15^,^16^. Ocean warming and acidification may also affect reef fishes via numerous mechanisms including reduced food for larvae, declining oxygen concentration due to warming, and changing behavioral patterns^17^,^18^,^19^. Even increased UV light could be a cause of benthic fish declines by reducing larval survival^20^.

The causes of coral loss are even more difficult to decipher, due to the context dependency of the many potential causal factors and the scarcity of data on nearly all of the putative drivers of coral mortality. Surprisingly, this is especially true for local stressors such as sedimentation, nutrient inputs and concentration, fishing intensity, and chemical contamination. Comprehensive monitoring in space and time of these basic parameters is scarce to non-existent for most tropical and subtropical coral reefs. In contrast, time-series of sea surface temperature on most reefs are readily available, indirectly via satellites, and directly through buoys, stations, and inexpensive data loggers. This has facilitated a large literature documenting the role of warming in driving coral loss, generally through coral bleaching and disease that lead to mortality^21^,^22^,^23^,^24^,^25^. This work has enabled the parameterization of global forecasts of bleaching, disease, and coral loss under different greenhouse gas emissions scenarios^26^,^27^,^28^. Paradoxically, in many ways we have a better understanding of (and evidence for) the effects of global factors like greenhouse gas emissions and ocean warming than of local impacts.

Given the lack of physical monitoring data, estimating the relative and interactive effects of local stressors is difficult to impossible in most locations. An alternative approach used to assess the role of local human impacts is to use isolated reefs as controls in comparison to presumably more impacted reefs closer to people^29^,^30^. This is based on the assumption that if local impacts (e.g., sedimentation, pollution, and fishing) are measurably affecting reefs, coral reef degradation should increase across sites as local human population density (and thus the magnitude of underlying local anthropogenic stressors) increases^29^. Numerous studies have documented clear relationships between reef isolation and reef fish abundance and trophic structure^3^,^31^,^32^, indicating that local human population density is a reliable predictor of fishing intensity^33^. Sedimentation and nutrient pollution is also related to human population density, given the role of human waste, coastal development, and erosion in these and other forms of pollution^10^,^34^ .Thus in response to the lack of cumulative local human stressors, live coral should be higher and the abundance of seaweeds should be lower on isolated reefs far from local human impacts. Although numerous studies have taken this approach to estimating the effect of combined local impacts to reefs, most past efforts suffer from very small sample sizes (e.g., a single surveyed reef as in^35^) or problems related to non-random site selection (e.g.,^36^ but see^37^).

The goal of this research was to estimate the contributions of local and global factors in coral reef degradation around the world (Fig. 1). Specifically, we determined whether coral declines and macroalgae increases (i.e., “reef degradation”) are correlated with reef “isolation”, which we defined as the number of people living within 50 km (i.e., more isolated reefs have few or no human inhabitants in close proximity). We focused on corals because they are the foundation species of reefs, creating both the larger tridimensional structure that provides “foundational” habitat and smaller-scale heterogeneity that reef inhabitants use primarily for refuge (to hide from predators) and foraging. Coral loss is thus a direct measure of habitat degradation. On some reefs where coral loss has been severe, the cover and biomass of fleshy and calcareous macroalgae has increased^38^. Therefore, macroalgal cover is an indirect measure of reef degradation. Moreover, in some cases, macroalgae can reduce the growth and survival of coral recruits thereby slowing coral population recovery from natural and anthropogenic disturbances^39^. Thus, macroalgal cover is often used as one measure of coral population recovery potential^40^ (i.e., “resilience”), although numerous other factors including predators, larval supply, and abiotic conditions also influence coral settlement and recruitment.

## Results and Discussion

Our results suggest that coral reef degradation is not correlated with human population density (Table 1, Fig. 2) and thus any impacts of local stressors were undetectable at a geographic scale. Most reefs are in close proximity to high human population densities, are exposed to numerous potential local stressors, and are directly exploited by people^33^. Thus, the absence of a signal of local impacts could be due either to their weak effects sizes or to an antagonistic interaction with global stressors. Although human population density was statistically significant in both global models (Table 1), it explained <1% of the among-reef variance in coral and macroalgal cover. This is not surprising, since our very large sample size enabled us to detect statistically significant but weak and ecologically meaningless relationships. This lack of a relationship was consistent within every region and subregion for which we had sufficient data (Tables S1 and S2, Figs. S2 and S3).

There is broad agreement that coral reefs in most regions continue to lose coral and generally degrade^4^,^5^. Yet there is ongoing debate about the proximate and ultimate causes of coral loss, particularly about the relative role of local and global factors^11^. There is a growing hope among coral reef scientists that local impacts are the dominant drivers of reef degradation and that these factors can be managed^41^. If true proximate threats could then be mitigated on site by local communities^40^. It is also assumed that local and global impacts are at least additive and likely synergistic^40^,^42^. This supposition underlies the widespread argument that human-dominated reefs can be made more resilient to global stressors (particularly warming) via local conservation and management^40^,^42^. Our results do not support either assumption.

This is the first global test of the hypothesis that isolated reefs are less degraded and have higher coral cover and less macroalgae cover. Most past tests of this hypothesis have relied on very small samples (i.e., <5), often based on non-random site selection. For example, Sandin *et al*.^36^ compared coral cover (and other community attributes) of coral reefs adjacent to four of the Northern Line Islands in the central Pacific that differed greatly in human population density (range 0–109 people/km of reef). They quantified earlier observations that coral cover was substantially greater adjacent to the two atoll islands with the fewest people (e.g., Kingman and Palmyra). However, our results indicate such patterns are not general: although some isolated reefs have exceptionally high coral cover, most do not (Fig. 3). In fact very isolated reefs with no human inhabitants within 50 km display a large range in coral cover and macroalgae cover, with a typical mean and distribution (Fig. 3 and S4). Our results are concordant with Smith *et al*.^37^ which tested the generality of the findings of Sandin *et al*.^36^ by surveying reefs surrounding 56 central Pacific islands. Their results indicated that coral and macroalgal cover were unrelated to the presence/absence of human inhabitants^37^.

Ocean warming is the most likely explanation for coral loss on isolated reefs. Anthropogenic warming due to greenhouse gas emissions causes coral mortality and population declines via coral bleaching and infectious diseases^21^,^23^,^43^. Warming and subsequent mass bleaching and coral mortality have been documented at countless isolated reefs, far from any local human influence in remote locations including Kirabati, Phoenix Islands, the Bahamas, the Chagos Archipelago, the outer Great Barrier Reef, and the northwest Hawaiian Islands^11^,^24^,^44^,^45^. A striking example is the mass-bleaching of hundreds of kilometers of the northern and central Great Barrier Reef– one of the world’s most isolated and well-protected reefs – earlier this year. Likewise, regional disease outbreaks, a primary cause of coral losses in regions including the Caribbean, have been linked to ocean warming^21^,^43^. Many scientists have noted the lack of any obvious association of coral disease outbreaks and mass bleaching episodes with proximity to people and urban centers^11^,^46^.

Many coral reef scientists assume that observed increases in macroalgae, though less common and far less severe than previously assumed^1^,^12^, are due to local impacts including generalized reduction of grazing pressure caused by the loss of key herbivores through disease and overfishing and by localized nutrient pollution^9^,^10^,^12^. This expectation is based on: 1) the observation that reefs with a greater abundance and diversity of herbivores tend to have less macroalgae^32^, and 2) the results of numerous small-scale experiments that increase nutrient concentration or exclude fishes generally find strong top-down and bottom-up control of macroalgae^47^,^48^. However, our results surprisingly indicate that macroalgal cover is not correlated with local human population density (Table 1, Fig. 2). Across 56 islands in the central Pacific, Smith *et al*.^37^ also found that macroalgal cover was unrelated to human presence and that reefs adjacent to densely populated islands such as Oahu, Hawaii had *less* macroalgae than many remote reefs far from human activities.

The causes of this unexpected global pattern are unclear. Perhaps increases in macroalgae are also caused by the global stressors^11^ that reduce coral populations and thus indirectly increase resource availability for benthic seaweeds and other organisms such as sponges and soft corals^2^,^49^. In this scenario, when and where herbivory is high relative to open space and benthic primary production, then macroalgal cover is low. Whereas when and where herbivory is low relative to open space, then macroalgal cover is high unless storms or other factors such as temperature extremes remove the seaweeds. This hypothesis is concordant with our results and the common observation that macroalgae often rapidly occupy available space directly following coral loss. If true, this finding has important management implications: fishing bans and reductions in coastal pollution, though desirable^11^, might not meaningfully reduce macroalgal abundance or restore corals if the ultimate drivers are larger-scale and beyond the control of local managers^50^. To be effective such local mitigation would need to be paired with reduction of the global stressors that have apparently enabled macroalgae to increase on some reefs. Alternatively, it is possible that predators, which are more abundant on isolated reefs^3^,^31^,^32^, suppress herbivores, either via direct consumption or behavioral modification that reduces foraging time, indirectly facilitating macroalgae^51^.

Our results also suggest that the effects of global and local stressors may be antagonistic and not additive or synergistic as widely assumed^40^,^42^. If the interaction were additive or multiplicative, coral populations exposed to both impact categories (i.e., those with high human population densities and ocean warming) would have lower coral cover. Antagonism, rather than synergism, could be due to co-tolerance of species to local and global stressors. If true, local stressors would reduce the abundance of species sensitive to global stressors, making locally disturbed communities less sensitive to large-scale factors like ocean warming^52^. This interpretation is consistent with numerous local, regional, and global studies indicating that local protection (e.g., the implementation of marine reserves), does not measurably lessen the impacts of ocean warming on coral populations^25^,^44^,^50^,^53^,^54^.

In conclusion, our findings contradict several widespread assumptions about the relative and interactive effects of local and global stressors causing coral losses around the world. We found that coral and macroalgal cover were not correlated with isolation from local anthropogenic stressors. Remote locations such as isolated reefs are often mythologized as pristine and barely impacted windows into the pre-human state of ecosystems^29^. In terms of fishes and other wildlife they can be, as reef fish biomass is clearly negatively correlated with human population density^3^,^31^,^32^ . But given the global reach of many other aspects of the human footprint^55^, perhaps it should not be surprising that coral losses on remote reefs match those on disturbed reefs adjacent to densely populated islands. The results of this and numerous other studies indicate that local management is unlikely to meaningfully increase the “resilience” of coral populations to warming, bleaching, disease, acidification, and other global stressors^25^,^44^,^50^,^54^. In fact, due to the apparent antagonistic relationship between local and global stressors, locally impacted reefs might be less sensitive to global stressors than isolated reefs^52^. Thus removing local stressors could counterintuitively *increase* sensitivity to warming of other large-scale disturbances^52^. Although our analysis did not detect a synergistic effect of localized human impacts, we believe there is adequate evidence in many locations to justify continued mitigation of small-scale stressors like overfishing and pollution. Given the continued global loss of reef-building corals and the results of this and other analyses indicating the primacy of large-scale stressors like warming^25^,^56^,^57^, the immediate, drastic reduction of greenhouse gas emissions is essential to restoring the health and functioning of coral reefs.

## Methods

### Coral reef survey data

Because coral cover (and other metrics of reef state) varies temporally (e.g., following disturbances and subsequent population recovery), one-time surveys of a small number of reefs would not be especially informative, in part because the observed population mean would not be a reliable estimate. To avoid this limitation we combined quantitative *in situ* surveys from 1708 reefs around the world, performed between 1996 and 2006 (1–15 m depth, mean depth; 7.1 m). Our approach, essentially a global space-for-time substitution with a very large sample size, enabled us to include reefs in a given region in various states of decline and recovery, and thus to more reliably estimate the population-level mean and distribution. Although presumably variable within and among regions, baseline coral cover is thought to be ~50–75% 58. Our survey data provides a snapshot of the degree of coral loss from the assumed baseline mean (e.g., a cover value of 25% would indicate an absolute 25–50% decline in cover).

Each survey quantified the percentage of the benthos occupied by living hard (scleractinian) corals and macroalgae. We defined macroalgae as “larger (canopy heights usually >10 mm), more rigid and anatomically complex algal forms” based on the definition of Steneck^59^, which includes erect calcifying species such as *Halimeda* spp. but not filamentous “turf” algae^59^. Benthic coverage was estimated either *in situ* by recording the number of points along 10–30 m transects that overlaid corals, macroalgae, etc. or from video and digital still images of the benthos. For each site, we only included the most recent survey available. Replicate cover measurements taken at different stations or depths on the same site were pooled into a single mean value. See Bruno *et al*.^38^ (including Appendix A) for a detailed description of data sources and procedures.

### Human population data

Human population counts estimated for the year 2005 were obtained from the Gridded Population of the World V.3 (GPWv3) at 0.25 degree resolution^60^. The GPWv3 consists of raster maps of human population density across the globe estimated every five years. We chose the year 2005 in our analysis because over 80% of the surveys were performed on or before this year. We did not use human population density for specific survey years because this information is not readily available for all reef sites. We calculated the maximum number of humans within 25 km, 50 km, and 100 km radius of each reef location using the package “*raster*” version 2.4 61 in the R statistical platform. In our final analysis, we chose human population density within 50 km radius of each reef location because it performed better during exploratory analysis (i.e., see Supplementary Information). In addition, human population density within 50 km has been useful to determine the impact of human activities in coral reefs^62^.

### Data analyses

**Figure 1:**
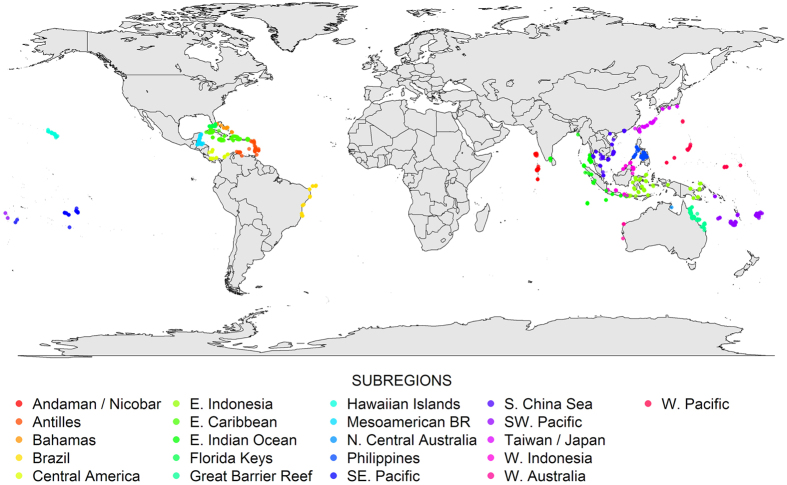
Location of reef sites by subregion. Map created in R 3.2.0 67, https://www.R-project.org.

**Figure 2:**
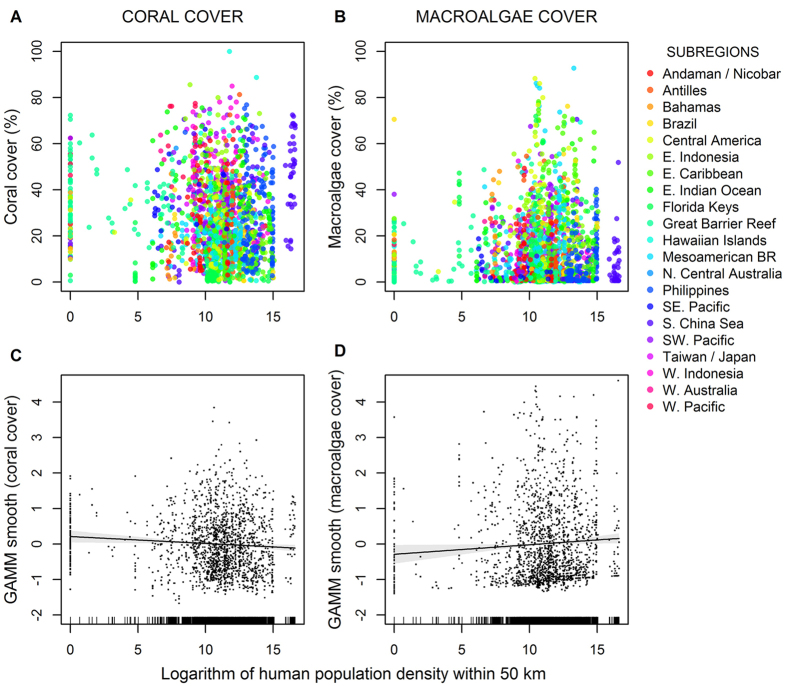
The global relationship between coral and macroalgae cover and the logarithm of human population density within 50 km of each reef location. Responses in figures (**A**,**B**) are raw coral and macroalgal cover data. Responses in (**C**,**D**) are smoothed with partial residuals from the generalized additive mixed effect models (GAMM, Table 1) to account for spatial autocorrelation. Human population density was used as a proxy for local impacts (e.g., fishing, development, and pollution). Colors correspond to different subregions. Subregional relationships and analyses are shown in Fig. S2 and S3 and Tables S1 ansd S2 respectively.

**Figure 3:**
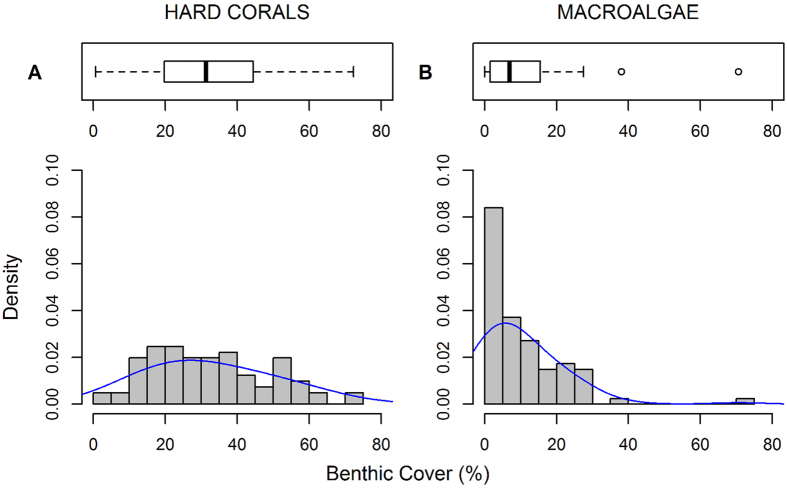
Density histograms of coral cover (**A**) and macroalgae cover (**B**) for sites with no human inhabitants within a 50 km radius. Average coral cover in these relatively isolated reefs was 32.5% (median 31.3% with 19.7–44.4%, 1^st^–3^rd^ quantile), slightly higher than the global average of 27.6% (median 24. 5% with 13.1–39.5% 1^st^–3^rd^ quantile). Mean macroalgal cover on these reefs (10.2%, median 6.9% with 1.6–15.5%, 1^st^–3^rd^ quantile) was somewhat lower than the global mean of 15.1% (median 10.0% with 3.1–22.4% 1^st^–3^rd^ quantile).

We used generalized additive mixed-effect models (GAMM)^63^ to analyze the relationships of coral and macroalgae cover with human population. A logit transformation was applied to the percent cover data and the logit was treated as normally distributed^64^ using a binomial family with a logit distribution. For each response variable (logit of coral or algae cover), a smoothness selection was fitted by maximum likelihood through the Laplace approximation. The log (x + 1) of human population density within 50 km of each reef location was used as the preferred predictor because it performed better than human population density within 25 km and 100 km during exploratory analysis. For both response variables we ran a global analysis and also analysis for ocean basins (i.e., Caribbean Sea, Pacific Ocean, and Indian Ocean) and subregions (Fig. 1). We used random intercept models where benthic cover within subregions was allowed to vary within ocean basin. To eliminate spatial autocorrelation observed in the raw data we added a correlation structure of the standard class autoregressive process of order 1 (corAR1) to each model. Potential spatial autocorrelation for each analysis was checked visually through spline correlogram plots of *lme* model residuals^65^ (Fig. S1). We performed model validation by assessing heterogeneity of the error distribution in the plot of normalized residuals against fitted values. For normality validation we used the normal scores of standardized residuals deviance^66^. All analyses were performed in R v.3.2.3 67 using the package *gamm4 *v.0.99–2 68 for GAMM, and the package *ncf* v.1.1–5 69 for spline correlograms.

### Data Availability

Data the analysis was based on is available at Dryad doi:10.5061/dryad.48r68 and code is available at GitHub https://github.com/johnfbruno/Bruno-and-Valdivia-Sci-Reports-2016.

## Additional Information

**How to cite this article**: Bruno, J. F. and Valdivia, A. Coral reef degradation is not correlated with local human population density. *Sci. Rep.* **6**, 29778; doi: 10.1038/srep29778 (2016).

## Supplementary Material

Supplementary Information

## Figures and Tables

**Table 1:** Results of the global generalized additive mixed model (GAMM) for coral and macroalgae cover in relation to the log of human population density within 50 km.

	Estimate	Std. Error	t-value	p-value	R-sq. (adj)
**Coral cover**					0.002350
Intercept	−0.937534	0.246593	−3.80194	0.00015	
s(log(human50 km + 1))	−0.063468	0.025273	−2.51130	0.01210	
**Macroalgae cover**					0.000425
Intercept	−1.828067	0.217381	−8.40949	0.00001	
s(log(human50 km + 1))	0.087640	0.039476	2.220080	0.02650	
