# Supplementary material for: Coral reef degradation is not correlated with local human population density
Source: Sci Rep. 2016 Jul 20;6:29778. doi: 10.1038/srep29778 (PMC4951719; doi:10.1038/srep29778)
Supplement: Supplementary Information [file srep29778-s1.pdf]

Supplementary Information for:

Coral reef degradation is not associated with local human population density

John F. Bruno[1] and Abel Valdivia[2]

[1] Department of Biology, The University of North Carolina at Chapel Hill, 340 Chapman Hall CB 3300, Chapel Hill, NC, USA 27599-3300

[2]Center for Biological Diversity, 1212 Broadway Street, Oakland, CA 94612

Text S1. The lack of a relationship between coral cover and human population density could be due to effective mitigation of local threats, to a degree that they are not measurably influencing coral populations. However, this seems unlikely. Alternatively, human population density may be a poor predictor of impacts caused by local activities, e.g., deforestation, sewage and other forms of pollution, etc. The intensity of some of these activities, at least in some locations, could be unrelated or even negatively related with the number of resident humans. However, numerous studies have documented clear relationships between reef isolation and reef fish abundance and trophic structure[3,45,46], indicating that local human population density is a reliable predictor of fishing intensity[47]. Sedimentation and nutrient pollution is also likely related to human population density, given the role of human waste, coastal development, and erosion in these and other forms of pollution[10,48] (a counter example is an island with relatively few people but extensive agriculture grown for export, e.g., oil palm plantations). Thus, based on this evidence it seems likely that human population density is a reasonably good proxy for local human impacts to the coastal landscape.

Table S1. Results of the regional general additive mixed models for coral and macroalgae cover in response to the log of human population density within 50 km.

REGION	Estimate	Std. Error	t-value	p-value	R-sq. (adj)
**Atlantic Basin**					
**Coral cover**					-0.00209
Intercept	-1.525092	0.178226	-8.557036	0.00000	
s(log(human50km+1))	-0.036832	0.035116	-1.048856	0.29470	
**Macroalgae cover**					0.003620
Intercept	-1.329713	0.130692	-10.17438	0.00000	
s(log(human50km+1))	0.084480	0.058782	1.437195	0.15120	
**Indian Basin**					
**Coral cover**					-0.01660
Intercept	-0.564253	0.331564	-1.701793	0.09150	
s(log(human50km+1))	0.0243978	0.222474	0.1096658	0.91290	
**Macroalgae cover**					-0.00476
Intercept	-2.181718	0.147070	-14.83455	0.00000	
s(log(human50km+1))	0.114048	0.132511	0.860672	0.39120	
**Pacific Basin**					
**Macroalgae cover**					-0.00726
Intercept	-2.081754	0.118082	-17.62975	0.00000	
s(log(human50km+1))	0.0971275	0.051521	1.8852110	0.05970	
**Coral cover**					-0.00270
Intercept	-0.647452	0.077045	-8.403597	0.00000	
s(log(human50km+1))	-0.083705	0.035996	-2.325400	0.02030	

Table S2. Results of the subregional general additive models for coral and macroalgae cover in response to the log of human population density within 50 km. edf: equivalent degrees of freedom; R-aq. (adj): adjusted R square; DE %: percent of null deviance explained.

**Subregion** (n = number of reefs)						
Coral/Macroalgae cover					**R-sq.**(adj)	**DE %**
Intercept	**Estimate**	**Std. Err.**	**z-value**	**Pr(>|z|)**		
Significance of Smooth	**edf**	**Ref. df**	**Chi-Sq**	**p-value**		
**Adaman/Nicobar** (n=37)						
Coral cover					-0.0126	1.74
Intercept	-1.1140	0.382	-2.9160	0.00354		
s(log(human50km+1))	1	1	0.1030	0.74800		
Macroalgae cover					-0.0285	0.01
Intercept	-2.1249	0.5325	-3.9900	6.6e-05		
s(log(human50km+1))	1	1	0.0000	0.98700		
**Antilles** (n=63)						
Coral cover					-0.00129	1.54
Intercept	-0.9796	0.2834	-3.4560	0.00055		
s(log(human50km+1))	1	1	0.1290	0.72000		
Macroalgae cover					-0.00582	1.6%
Intercept	-1.8510	0.3688	-5.0190	5.19e-07		
s(log(human50km+1))	1	1.0010	0.1630	0.686		
**Bahamas** (n=16)						
Coral cover					-0.0143	8.43
Intercept	-1.929	0.766	-2.5180	0.01180		
s(log(human50km+1))	1	1	0.1030	0.74800		
Macroalgae cover					-0.0502	2.58
Intercept	-1.3016	0.6139	-2.1200	0.034		
s(log(human50km+1))	1	1	0.0810	0.776		
**Brazil** (n=45)						
Coral cover					0.0031	4.61
Intercept	-1.2951	0.3644	-3.554	0.00038		
s(log(human50km+1))	1	1	0.2170	0.64100		
Macroalgae cover					0.0284	5.13
Intercept	-1.3520	0.3735	-3.6200	0.00029		
s(log(human50km+1))	1	1	0.4390	0.508		
**Central America** (n=56)						
Coral cover					-0.00462	1.14
Intercept	-1.1521	0.3132	-3.6790	0.00024		
s(log(human50km+1))	1	1	0.0640	0.80000		
Macroalgae cover					0.157	17.9
Intercept	-0.9239	0.3012	-3.0680	0.00215		
s(log(human50km+1))	1.6880	2.065	1.9720	0.38500		
**Eastern Indonesia** (n=140)						

Coral cover					0.0412	4.6%
Intercept	-0.5260	0.1754	-2.999	0.00271		
s(log(human50km+1))	1	1	0.7330	0.39200		
Macroalgae cover					0.0317	4.68
Intercept	-1.7997	0.2437	-7.3480	1.53e-13		
s(log(human50km+1))	1	1	0.9310	0.33500		
**Eastern Caribbean** (n=144)						
Coral cover					-0.00503	0.19
Intercept	-1.601	0.223	-7.1790	7.04e-13		
s(log(human50km+1))	1	1	0.0200	0.88700		
Macroalgae cover					-0.00408	0.28
Intercept	-0.9975	0.1879	-5.308	1.11e-07		
s(log(human50km+1))	1	1	0.102	0.7490		
**Eastern Indian Ocean** (n=80)						
Coral cover					-0.00366	0.91
Intercept	-0.5846	0.2332	-2.506	0.0122		
s(log(human50km+1))	1.0640	1.1240	0.009	0.9450		
Macroalgae cover					-0.0035	1.09
Intercept	-2.2047	0.3755	-5.8720	4.31e-09		
s(log(human50km+1))	1	1	0.1440	0.70400		
**Florida Keys** (n=158)						
Coral cover					-0.0064	0.00
Intercept	-2.4276	0.2914	-8.330	<2e-16		
s(log(human50km+1))	1	1	0	0.992		
Macroalgae cover					0.0211	2.57
Intercept	-1.7741	0.2267	-7.8250	5.07e-15		
s(log(human50km+1))	1.1490	1.2830	0.4200	0.623		
**Great Barrier Reef** (n=150)						
Coral cover					0.0593	6.28
Intercept	-0.7908	0.1772	-4.464	8.05e-06		
s(log(human50km+1))	1	1	1.198	0.274		
Macroalgae cover					0.0446	5.51
Intercept	-2.1275	0.2694	-7.898	2.83e-15		
s(log(human50km+1))	1	1	1.16	0.281		
**Hawaiian Islands** (n=37)						
Coral cover					-0.0258	0.25
Intercept	-0.9301	0.3652	-2.547	0.0109		
s(log(human50km+1))	1	1	0.025	0.874		
Macroalgae cover					-0.0284	0.01
Intercept	-2.4407	0.6056	-4.03	5.57e-05		
s(log(human50km+1))	1	1	0.001	0.976		
**Mesoamerican Barrier** (n=150)						
Coral cover					0.00179	0.94
Intercept	-1.3571	0.2025	-6.703	2.05e-11		

s(log(human50km+1))	1	1	0.088	0.767		
Macroalgae cover					0.0634	7.07
Intercept	-1.331	0.204	-6.523	6.88e-11		
s(log(human50km+1))	1	1	1.98	0.159		
**Philippines** (n=167)						
Coral cover					0.0063	1.12
Intercept	-0.9565	0.1730	-5.528	3.25e-08		
s(log(human50km+1))	1	1	0.332	0.564		
Macroalgae cover						
Intercept	-2.3441	0.2835	-8.269	<2e-16	0.13	12.4
s(log(human50km+1))	1	1	1.917	0.166		
**Southeast Pacific** (n=32)						
Coral cover					-0.0314	0.17
Intercept	-0.7441	0.3784	-1.967	0.0492		
s(log(human50km+1))	1	1	0.007	0.935		
Macroalgae cover					0.0208	5.37
Intercept	-2.8502	0.7853	-3.629	0.0003		
s(log(human50km+1))	1	1	0.076	0.783		
**South China Sea** (n=102)						
Coral cover					0.109	10.8
Intercept	-0.4170	0.2045	-2.039	0.0414		
s(log(human50km+1))	1	1	1.794	0.1800		
Macroalgae cover					-0.009	0.06
Intercept	-2.3154	0.3463	-6.686	2.29e-11		
s(log(human50km+1))	1	1	0.008	0.9270		
**Southwest Pacific** (n=161)						
Coral cover					0.00248	0.87
Intercept	-0.8208	0.1712	-4.793	1.64e-06		
s(log(human50km+1))	1	1	0.188	0.665		
Macroalgae cover					0.0113	1.87
Intercept	-1.5510	0.2081	-7.454	9.06e-14		
s(log(human50km+1))	1	1	0.286	0.5930		
**Taiwan-Japan** (n=50)						
Coral cover					-0.0156	0.50%
Intercept	-0.7443	0.3028	-2.458	0.014		
s(log(human50km+1))	1	1	0.031	0.861		
Macroalgae cover					-0.015	0.58%
Intercept	-1.8193	0.4086	-4.453	8.47e-06		
s(log(human50km+1))	1	1	0.031	0.861		
**West Indonesia** (n=71)						
Coral cover					0.0138	2.67%
Intercept	-0.4822	0.2449	-1.969	0.049		
s(log(human50km+1))	1	1	0.35	0.554		
Macroalgae cover					-0.0036	1.2%

Intercept	-2.3858	0.4289	-5.562	2.67e-08		
s(log(human50km+1))	1	1	0.083	0.774		
**Western Pacific** (n=44)						
Coral cover					0.0414	5.95%
Intercept	-0.1443	0.3035	-0.475	0.635		
s(log(human50km+1))	1	1	0.341	0.559		
Macroalgae cover					-0.0231	0.06%
Intercept	-1.6516	0.4103	-4.025	5.69e-05		
s(log(human50km+1))	1	1	0.002	0.965		

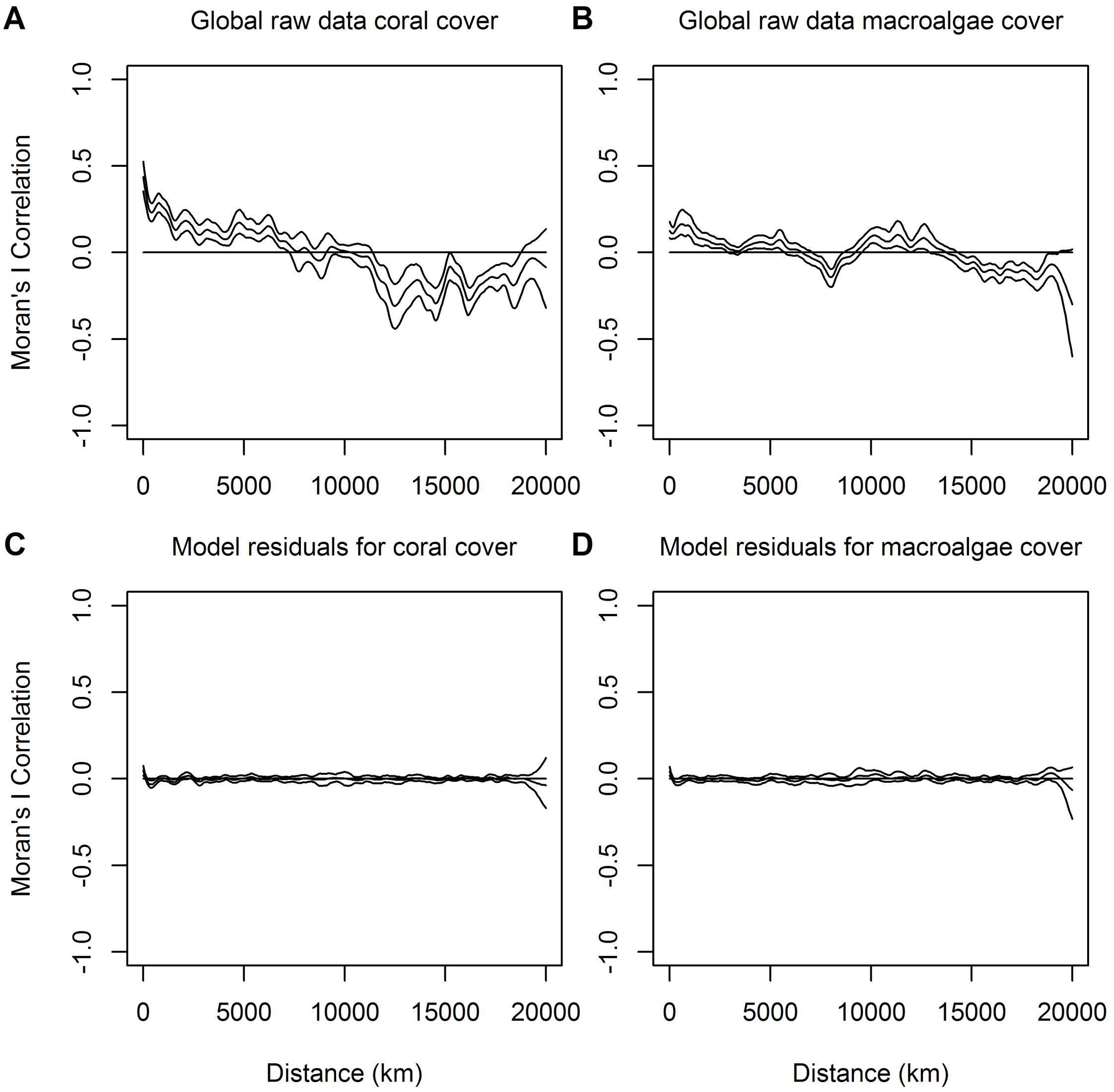

Figure S1. Global spline correlograms for the raw data of coral cover (A), macroalgae cover (B), and the residuals of the generalized additive mixed model for coral cover (C), and macroalgae cover (D) after accounting for spatial autocorrelation by including ocean basin and subregion as random factor and an autoregressive correlation structure (corAR1).

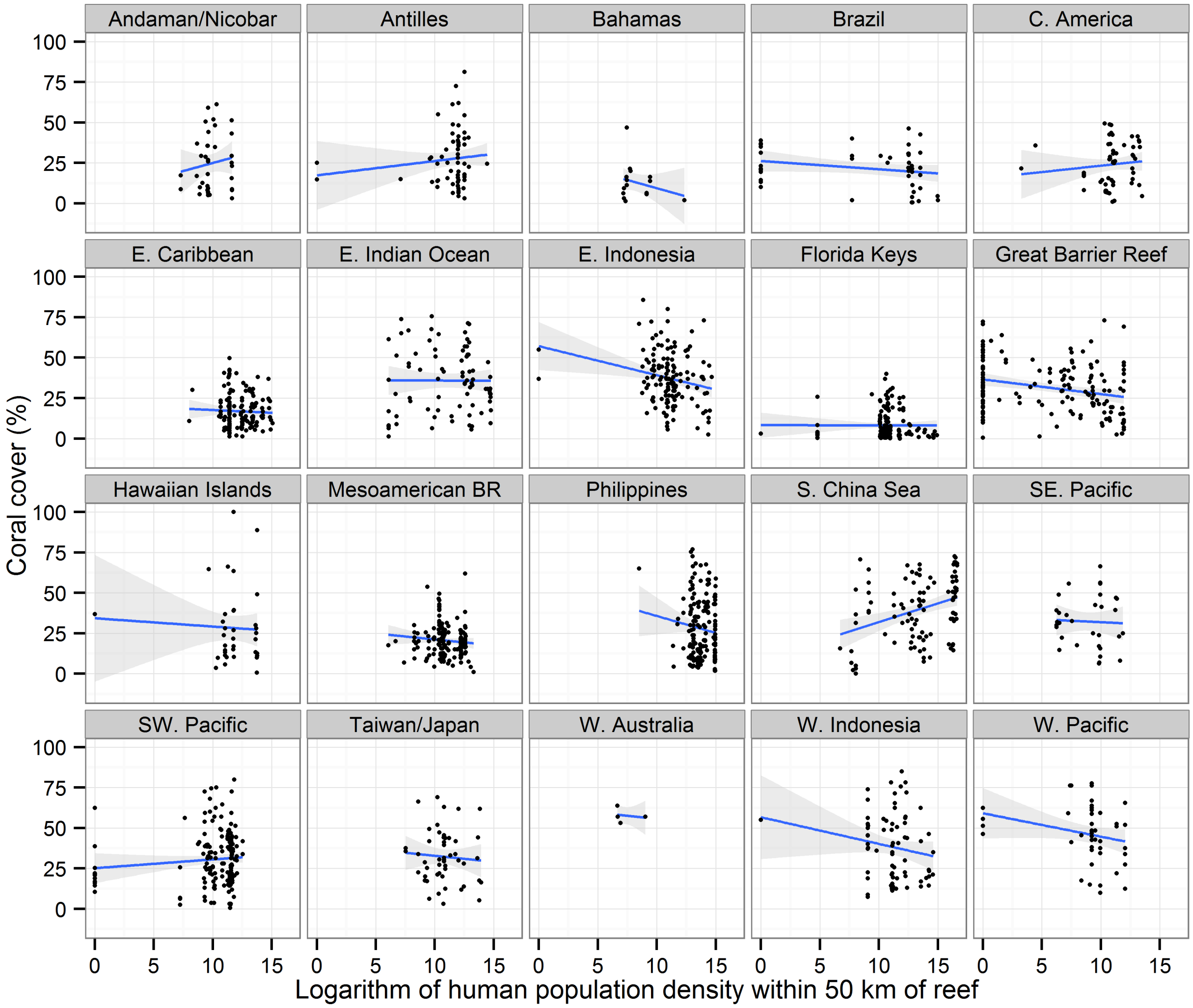

Figure S2. Subregional relationship between coral cover and the logarithm of human population density within 50 km of each reef location. Trend lines are the generalized additive models (GAM) smoothing and 95% confidence interval. Results of the GAM analyses for each subregion are shown in Table S2.This relationship was not analyzed in reefs within West Australia because only four sites were surveyed.

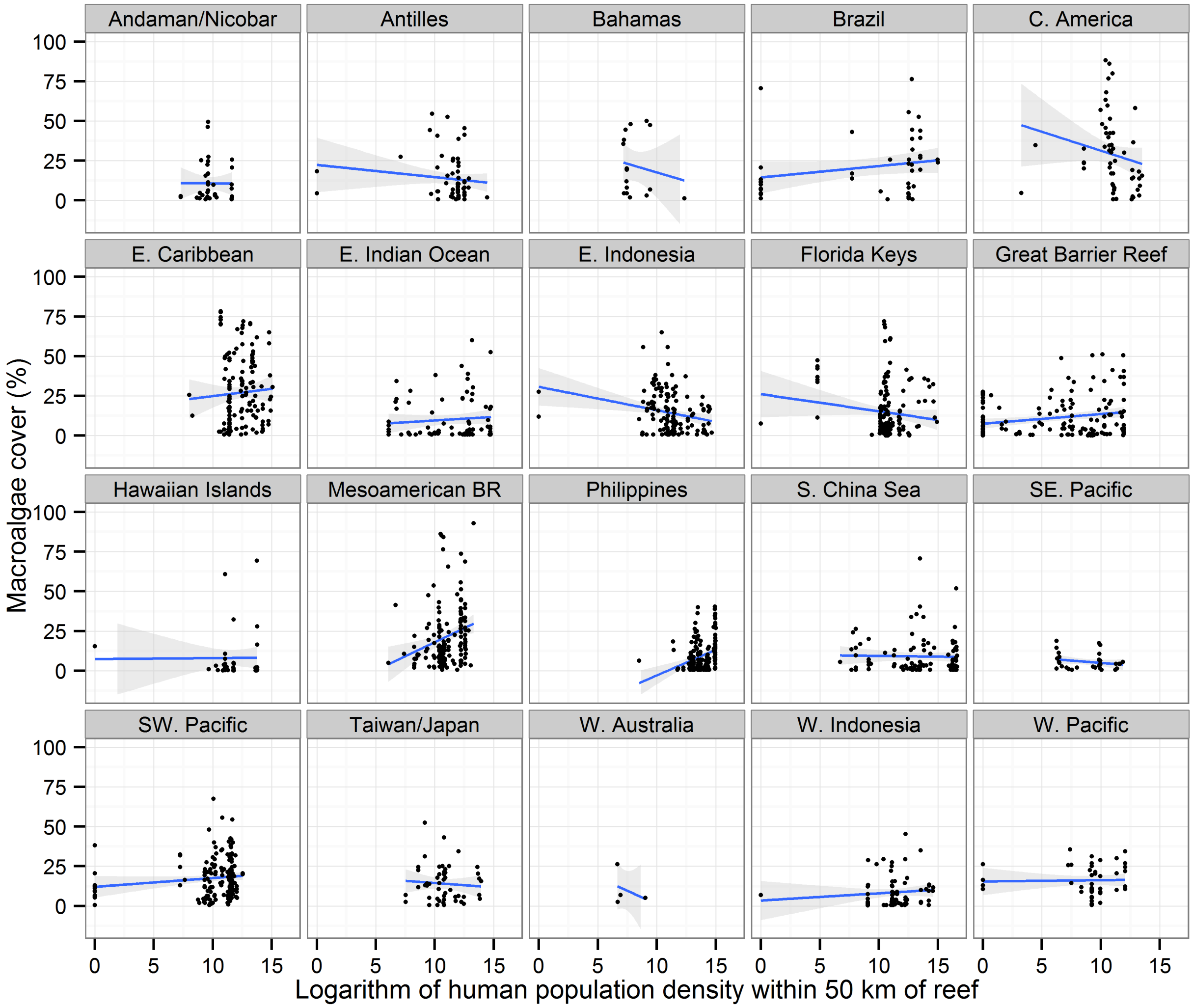

Figure S3. Subregional relationship between macroalgae cover and logarithm of human population density within 50 km of each reef location. Trend lines are the generalized additive models (GAM) smoothing and 95% confidence interval. Results of the GAM analysis for each subregion are shown in Table S2. This relationship was not analyzed in reefs within West Australia because only four sites were surveyed.

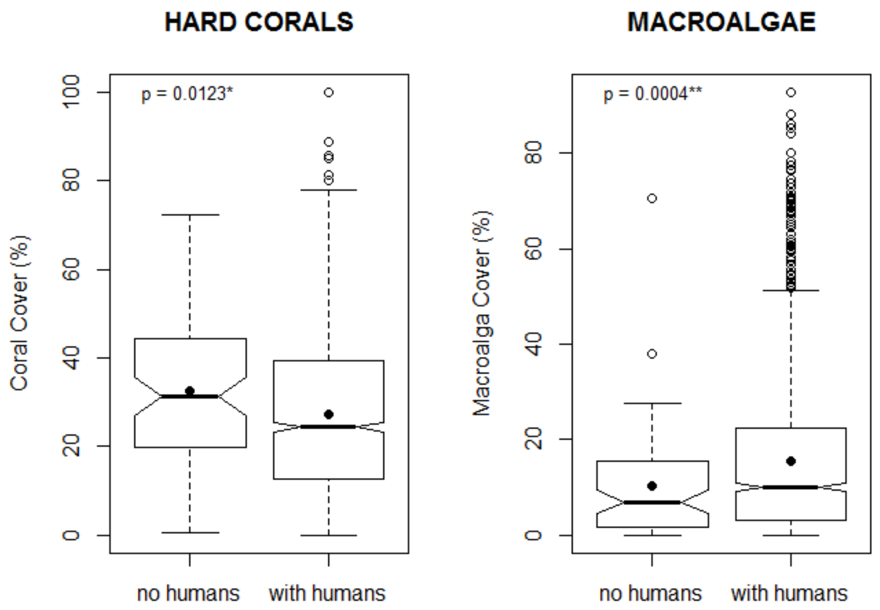

Figure S4. Global comparison of absolute cover of coral and macroalgae on reefs without (n = 81) and with (n = 1627) resident humans within a 50 km radius of the reef site. Coral cover is slightly higher and macroalgal cover is slightly lower on reefs without humans. This could be due to local human impacts and might represent a small but realized average effect (~5%) of any human presence on coral and macroalgal cover. However, these differences are more likely due to underlying geophysical differences between inhabited and uninhabited islands. For example, most reefs with no human residents were islands within Brazil, the Great Barrier Reef, and the West and Southwest Pacific subregions (see Fig. S3 and Fig 1). These reefs are adjacent to very small, marginal islands or atolls that are generally not habitable by humans due to lack of fresh water and resources. They also typically lack rivers and streams, substantial terrestrial organic soil and vegetation, and topographic complexity. Therefore terrigenous inputs that naturally greatly influence numerous geochemical aspects of nearshore reefs are absent. In short, human presence is confounded with reef type and abiotic environmental characteristics known to strongly influence reef communities. It is impossible to tease the two apart and make strong inferences about the local impacts of humans by comparing reefs adjacent to small, uninhabited atolls/islands to reefs close to large volcanic islands or continental land masses.
